# Radiation Induced Lymphopenia Is Associated With the Effective Dose to the Circulating Immune Cells in Breast Cancer

**DOI:** 10.3389/fonc.2022.768956

**Published:** 2022-04-28

**Authors:** Fang Chen, Jian-Yue Jin, Timothy S.K. Hui, Haiman Jing, Hong Zhang, Yaqing Nong, Ying Han, Weili Wang, Lingyu Ma, Fan Yi, Qingqing Chen, Yongsheng Zhang, Pingfu Fu, Li Yang, Zhiyuan Xu, Feng-Ming Spring Kong

**Affiliations:** ^1^Department of Clinical Oncology, The University of Hong Kong-Shenzhen Hospital, Shenzhen, China; ^2^Department of Clinical Oncology, Hong Kong University Li Ka Shing Medical School, Hong Kong, Hong Kong SAR, China; ^3^Department of Radiation Oncology, Case Western Reserve University, Cleveland, OH, United States; ^4^Department of Radiation Oncology, University of Maryland School of Medicine, Baltimore, MD, United States; ^5^Department of Population and Quantitative Health Sciences, Case Western Reserve University, Cleveland, OH, United States

**Keywords:** lymphopenia, radiation, effective dose to the circulating immune cells (EDIC), prediction model, breast cancer

## Abstract

**Background:**

Lymphopenia is a known significant factor for treatment outcome in cancer patients, with underlying risk factor poorly understood in breast cancer. We hypothesize that the effective dose to the circulating immune cells (EDIC) which was related with lymphopenia in lung cancer will also have significant effect for radiation induced lymphopenia (RIL) in patients with breast cancer.

**Material and Methods:**

Patients treated with adjuvant radiotherapy (RT) and with complete blood tests within one week from RT end/start (post/preRT) were eligible in this study. Radiation dosimetric factors were collected retrospectively, and EDIC for each patient was calculated based on the doses to lung, heart and total body according to the model description, as previously reported. RIL was defined by the CTCAE5.0 based on postRT peripheral lymphocyte count (PLC). Linear regression was first used to test the correlation between EDIC with post/preRT PLC ratio and postRT PLC, using all these as continuous variables. Normal tissue complication probability (NTCP) was used to develop models that predict the CTCAE graded RIL from EDIC.

**Results:**

A total of 735 patients were eligible. The mean post/preRT PLC ratio was 0.66 (95% CI: 0.64-0.68) and mean EDIC of breast cancer was 1.70Gy (95% CI: 1.64-1.75). Both post/preRT PLC ratio and postRT PLC were significantly correlated with EDIC (P<0.001), with R^2^ of 0.246. For patients with normal preRT PLC, the post/preRT PLC ratio was better associated with EDIC, and postRT PLC was expressed as PLC*_preRT_* × (0.89 – 0.16 × *EDIC*). For patients with preRT lymphopenia, postRT PLC was better associated with EDIC and it was 1.1 – 0.17 × *EDIC*. Using binned EDIC as the dose variable, the bootstrap validated NTCPs fit the data nicely with R^2^ of 0.93, 0.96, and 0.94 for grade-1, grade-2, and grade-3 RIL, respectively. The corresponding EDIC to induce 50% of grade-1, grade-2 and grade-3 RIL was 1.2, 2.1 and 3.7 Gy, respectively.

**Conclusion:**

EDIC is a significant factor for RIL in patients with breast cancer, and may be used to compute the risk of lymphopenia in each individual patient with the use of the conventional NTCP modeling. External validation is needed before the EDIC can be used to guide RT plan.

## Introduction

The immune system is critical for the development and management of malignant tumors. Lymphocyte is a major component of the immune system while lymphopenia was reported to be associated with poor prognosis in patients with breast cancer and other malignant cancers (1–4). However, lymphocytes are extremely radiosensitive (5, 6) and exposure to as low as 1 Gy of radiation can destroy mature circulating lymphocytes (7). Therefore, radiotherapy (RT) can damage the immune system and cause potential immunosuppression while it is applied for its role on killing malignant tumor cells.

Radiation induced lymphopenia (RIL) is common and known as a negative prognostic factor in many types of malignant solid tumors (8–15). In breast cancer, we have demonstrated that 60.5% patients had lymphopenia and 92.7% patients had some degree of reduction in peripheral lymphocyte count (PLC) after adjuvant RT (16). RIL or the ratio of nadir PLC and pretreatment PLC were also found to be a potential predictor for ipsilateral breast tumor recurrence or 5-year disease-free survival in breast cancer (17, 18). Meanwhile, several phase III clinical trials involving immunotherapy showed positive results which have significantly changed the treatment strategy for triple negative breast cancer (19, 20). The keynote 522 study (20) had enrolled previously untreated stage II or stage III triple-negative breast cancer and most of them would receive adjuvant RT concurrent with pembrolizumab after surgery. The effect of immunotherapy might be weakened by RIL since patients with low PLC had lower immune response rate while receiving checkpoint inhibitors (21). Therefore, it is important to establish a prediction model of RIL to improve RT plan of a better lymphocyte-sparing technique to reduce treatment-related lymphopenia.

Studies had revealed that radiation dose to the lymphatic system played an important role on RIL (6, 12). Our prior study also found that several radiation dosimetric factors (such as mean lung dose) were significant risk factors for lymphopenia after RT in patients with breast cancer (16). A simple RIL model based on radiation dose to the lymphatic system will be helpful clinically to improve RT plan and minimize lymphopenia. The lymphatic system is a complicated system composed of lymphoid organs such as lymph nodes/ducts, bone marrow and thymus, non-lymphoid organs such as lung and liver where lymphocytes reside, and circulation blood which transport lymphocytes to different parts of the body (22). Here we hypothesized that radiation dose to the lymphocytes in circulation blood to be critical for RIL because it was observed in breast and brain radiation patients while there is little lymphoid tissue in radiation of these regions (13, 23, 24). In addition, RIL is directly measured according to the number of lymphocytes in a unit volume of blood, and the active lymphocytes in circulation blood may be more radiosensitive than non-active lymphocytes in other parts of the lymphatic system (5, 6).

However, it is difficult to determine the radiation dose to the lymphocytes in circulation blood because they are moving targets. We have previously developed a model for the effective dose to the circulating immune cells (EDIC) for thoracic radiation based on planning dosimetric data, and demonstrated that EDIC was associated with both overall survival (OS) and local progression free survival (LPFS) after thoracic radiotherapy in non-small cell lung cancer (NSCLC) patients (25). This EDIC model has been validated externally in NSCLC (26) and esophageal cancer (15, 27), and further extended to abdominal radiation with consideration of other immune organs beyond circulating blood, including spleen, lymphatic ducts and bone marrows (22). Because the main irradiated blood-containing structures of breast cancer are similar to that of other thoracic malignancies, we hypothesized that this modeled EDIC may also be applicable to breast cancer. The purpose of this study is two folds: 1) to investigate whether EDIC is associated with RIL in patients with breast cancer; 2) to develop a normal tissue complication probability (NTCP) model for RIL by considering the EDIC as the dose to the circulating lymphocytes, similar as the conventional NTCP model for other normal structures (such as NTCP of radiation induced pneumonitis versus lung dose).

## Materials and Methods

### Study Population

Patients with breast cancer who had received adjuvant radiation between March 2015 to February 2020 in the University of Hong Kong-Shenzhen Hospital were enrolled in this study. Inclusion criteria: pathology confirmed invasive breast cancer, aged 18-year-old and above, underwent adjuvant radiation therapy. Exclusion criteria: non-invasive breast cancer, recurrent or stage IV breast cancer, breast lymphoma, immune related diseases, without PLC within 7 days before and after radiation in the University of Hong Kong-Shenzhen Hospital.

### Data Collection

Following information of patients were collected: 1) radiation dosimetric factors including RT technique, RT fields, RT fractionations, mean heart dose (MHD), mean lung dose (MLD), and integral dose, which has a unit of dose*volume (to avoid confusion, here we referred it as integral total dose volume [ITDV]); 2) Other clinical factors including age, tumor laterality, ER/PR/HER-2 subtype, stage, surgical approaches, chemotherapy, endocrine therapy, target therapy; 3) PLC within 7 days at the beginning and end of radiation. Lymphopenia was graded by Common Terminology Criteria for Adverse Events (CTCAE) version 5.0. Lymphopenia was defined as PLC cut-off of 1.06 × 10^9^ in our institution. Grade 1, 2, 3 and 4 lymphopenia were defined as PLC cut-off of 1.06-0.8 × 10^9^, 0.8-0.5 × 10^9^, 0.5-0.2 × 10^9^ and 0.2 × 10^9^/L, respectively. The study endpoints were numerical value of post/preRT PLC ratio, and postRT lymphopenia graded by CTCAE5.0 based on postRT PLC.

### EDIC Calculation

A recently reported EDIC model was used for this study (25). The details of the EDIC model derivation have been described previously and validated externally (22, 25). Basically, the model derivation includes 4 steps: 1) convert the mean dose of each single blood containing organ (such as lung and heart) in one fraction into blood dose and volume by considering continuous blood flow through the organ during the time of radiation delivery (22, 25); 2) estimate the fractionation effect by considering that the irradiated blood uniformly mixed with un-irradiated blood after each fraction (13); 3) convert the blood dose volume data modified by the fractionation effect into a blood equivalent uniform dose; and 4) finally EDIC is the sum of the blood equivalent uniform doses (EUDs) contributed by each blood-containing organs, including lung, heart, large and small blood vessels with an assumption that large and small vessels are uniformly distributed in the body. The model is finally expressed as $EDIC=12\%\times MLD+8\%\times MHD+\left[45\%+35\%\times 0.85\times {\left(\frac{n}{45}\right)}^{\frac{1}{2}}\right]\times ITDV/\left(61.8\times 10^{3}\right)$ , where MLD, MHD, and ITDV are the mean total lung dose, mean heart dose and integral dose (or integral total dose volume), respectively, and n is the number of radiation fractions (25). The ITDV was calculated as the mean external contour dose multiplying with the volume of the external contour. The CT scan region was from the lower jaw to L1 vertebra in this study.

### Statistical Method

The effects of potential risk factors of RIL (post/preRT PLC ratio) were estimated using univariate analysis initially and further estimated using multivariable model. To avoid the unstable and inaccurate estimates of the coefficients, the stepwise linear regression based on the Akaike information criterion minimum was used to select variables for inclusion in the multivariate analysis. Meanwhile, the collinearity testing was performed using the variance inflation factor (VIF), and VIF > 10.0 was interpreted as indicating multicollinearity. Variables with VIF > 10.0 were not included in the final model. Coefficients and corresponding 95% confidence interval were calculated for the linear regression model. Another linear regression was used to investigate the numerical correlation between the post/preRT PLC ratio (or postRT PLC) with EDIC. Statistical analysis was performed using R software (version 3.6.2; https://www.R-project.org).

To develop the NTCP model for RIL, the Solver program in Excel was used to fit the clinical data into the NTCP model. The patients were binned into the following 8 groups according to their EDIC values: 1) EDIC <1.0; 2) 1≤ EDIC<1.5; 3) 1.5≤ EDIC<2.0; 4) 2.0≤ EDIC<2.5; 5) 2.5≤ EDIC<3.0; 6) 3.0≤ EDIC<3.5; 7) 3.5≤ EDIC<4.0; and 8) EDIC≥4.0. The average EDIC in each bin was calculated. The percentage of patients had Grade 1+, Grade 2+ and Grade 3+ lymphopenia at each bin was also calculated, respectively. The calculated percentage of patients with lymphopenia was considered as clinically observed NTCP for lymphopenia, and NTCP versus average EDIC was then fitted with the NTCP model expressed as


(1) $$NTCP=1/\left[1+{\left(\frac{D50}{EDIC}\right)}^{k}\right]$$

where D50 and k are fitting parameters. When the value of EDIC that generated NTCP of 50% was considered to be D50. For better comparison, we also used the original binary data for each patient to fit the NTCP model. Bootstrap validations were also performed for both binning and original binary data settings. Internal generalizability was evaluated over 1000 bootstrap samples, which were obtained by selecting patients randomly from the study cohort, with replacement to produce datasets having the same number of patients as the original cohort. Confidence intervals (CIs) for fit parameters were calculated using the bias corrected and accelerated bootstrap (BCa) method.

## Results

### Characteristics of Patients and Radiation Dosimetric at Baseline

Between March 2015 to February 2020, 1559 patients with breast cancer received adjuvant RT in the University of Hong Kong-Shenzhen Hospital. Among them, 735 patients with invasive breast cancer were eligible for this study **(** **Supplementary Figure 1** **for patient selection)**. **Table 1** lists the characteristics of patient, tumor, pre-radiation treatment factors and radiation dosimetric factors at baseline. The median age was 45 years (range 26-86). The mean doses of lung, heart and integral body were 5.5Gy (95% CI: 5.3-5.6), 2.4Gy (95% CI: 2.2-2.5) and 4.4Gy (95% CI: 4.3-4.5), respectively.

**Table 1:** Patient characteristics, dosimetric factors and their predictive values on radiation induced lymphopenia (post/preRT PLC ratio).

	n (%)/Mean (95%CI)	Univariate analysis		Multivariable analysis	
		Coefficient (95%CI)	p value	Coefficient (95%CI)	p value
Age [median (range)]-year	45 (26-86)	-0.001 (-0.003, 0.001)	0.355		
Tumor side (left vs non-left)^†^	371(50.5%) vs 364 (49.5%)	-0.035 (-0.069, -0.001)	0.046		
Tumor stage^*^					
IA/IB	193 (26.2%)	0			
IIA/IIB	332 (45.2%)	-0.061 (-0.100, -0.021)	0.003		
IIIA/IIIB/IIIC	210 (28.6%)	-0.224 (-0.268, -0.181)	<0.001		
Node status (N0 vs N+)	290 (39.5%) vs 445 (60.5%)	-0.139 (-0.172, -0.105)	<0.001		
BCT vs Mastectomy	373 (50.7%) vs 362 (49.3%)	0.105 (0.071, 0.138)	<0.001		
SLNB vs ALND	265 (36.1%) vs 470 (63.9%)	0.135 (0.100, 0.170)	<0.001		
Chemotherapy (none vs yes)	69 (9.4%) vs 666 (90.6%)	0.008 (-0.051, 0.067)	0.800		
Target therapy (non vs yes)	556 (75.6%) vs 179 (24.4%)	-0.019 (-0.059, 0.022)	0.364		
Endocrine therapy (none vs yes)	186 (25.3%) vs 549 (74.7%)	-0.004 (-0.044, 0.035)	0.837		
RT technology					
RapidArc	123 (16.7%)	0		0	
2D-fields	277 (37.7%)	0.351 (0.307, 0.395)	<0.001	0.176 (0.078, 0.275)	<0.001
3DCRT	335 (45.6%)	0.268 (0.225, 0.311)	<0.001	0.146 (0.069, 0.223)	<0.001
EDIC (95% CI)—Gy	1.7 (1.6-1.8)	-0.156 (-0.177, -0.136)	<0.001	-0.106 (-0.156, -0.055)	<0.001
RT fields (breast vs breast/chestwall + regional LNs)	277 (37.7%) vs 458 (62.3%)	-0.153 (-0.187, -0.119)	<0.001		
RT Dose (40.5Gy vs 50Gy)	665 (90.5%) vs 70 (9.5%)	-0.109 (-0.167, -0.051)	<0.001	0.056 (-0.009, 0.120)	0.091
Use of breathing control (none vs yes)	721 (98.1%) vs 14 (1.9%)	-0.202 (-0.327, -0.077)	0.002		
RT fractions (15 vs 25)	665 (90.5%) vs 70 (9.5%)	-0.109 (-0.167, -0.051)	<0.001		
Mean heart dose (95% CI)—Gy	2.4 (2.2-2.5)	-0.036 (-0.044, -0.029)	<0.001		
Heart dose_Dmax (95% CI)—Gy	26.5 (25.2-27.9)	-0.001 (-0.002, -0.0001)	0.026	0.001 (0.0002, 0.002)	0.012
Mean dose of the total body (95% CI)—Gy	4.4 (4.3-4.5)	-0.069 (-0.078, -0.060)	<0.001		
V5 of bilateral lungs (95%CI)—Gy	22.3 (21.3-23.2)	-0.009 (-0.010, -0.008)	<0.001		
V20 of bilateral lungs (95%CI)—Gy	10.2 (9.9-10.5)	-0.017 (-0.021, -0.013)	<0.001		
Mean bilateral lung dose (95%CI)—Gy	5.5 (5.3-5.6)	-0.046 (-0.053, -0.040)	<0.001		

RT, radiotherapy; LN, lymph nodes; BCT, Breast conserving therapy; SLNB, Sentinel lymph node biopsy; ALND, Axillary lymph node dissection; EDIC, effective dose to the circulating immune cells); V5 (20), relative volume receiving more than 5Gy (20Gy).

^†^Non-left included 363 right and 1 bilateral breast cancer.

^*^Tumor stage was identified as the higher stage between clinical and pathological stage for patients who had received neoadjuvant chemotherapy and was identified as pathological stage for patients who had upfront surgery.

### Correlation Between EDIC and postRT/preRT PLC (or postRT PLC)

Overall, 92.7% patients had some degree of reduction in PLC postRT, and 60.5% (445/735) patients had lymphopenia postRT (**Supplementary Table 1**). The mean post/preRT PLC ratio was 0.66 (95% CI: 0.64-0.68). The mean EDIC of breast cancer was 1.70Gy (95% CI: 1.64-1.75). Univariate and multivariable regression analyses showed that EDIC was one of the significant risk factors (P<0.001) for lymphopenia (post/preRT PLC ratio) (**Table 1**).

**Figure 1:**
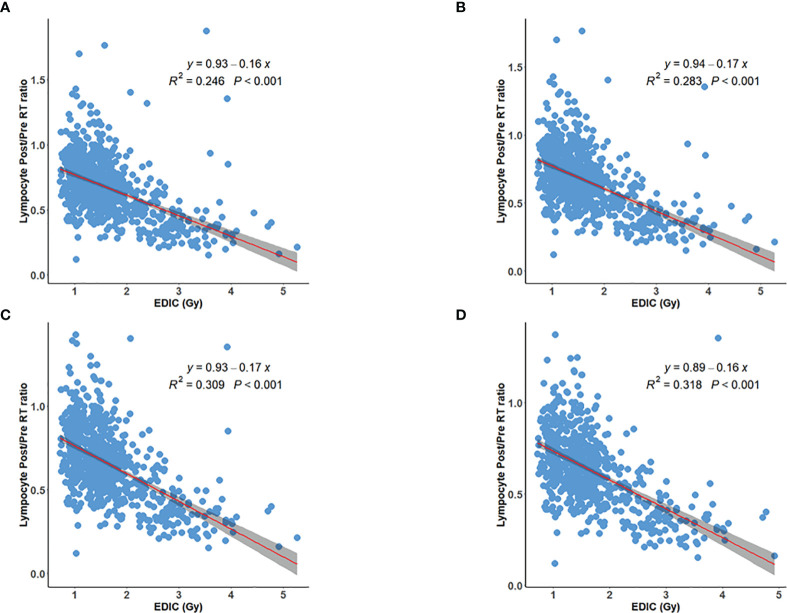
Inverse linear relation between post/preRT PLC Ratio and EDIC for **(A)** all 735 patients; **(B)** 732 patients excluding 3 patients with grade 3 preRT lymphopenia; **(C)** 714 patients excluding additional 18 patients with grade 2 preRT lymphopenia; **(D)** 630 patients excluding additional 84 patients with grade 1 preRT lymphopenia.

Linear regression showed that the post/preRT PLC ratio decreased with increasing EDIC (R^2^ = 0.246, p<0.001) (**Figure 1A**). However, we noted that a significant number of patients were outliers that not fit to the linear regression. Interestingly, most of these outlier patients had baseline (preRT) lymphopenia (or low preRT PLC), which may had confounded the radiation effect. In this study, 14.3% (105/735, 95%CI: 14.2-14.3%) patients had CTCAE5.0 defined lymphopenia before RT (11.4%, 2.5%, 0.4% and 0 for grade 1, 2, 3 and 4, respectively) **(** **Supplementary Table 1**). As shown in **Figures 1B–D**, the correlation represented by R^2^ was improved from 0.246 to 0.283 by excluding 3 patients with grade-3 baseline lymphopenia, improved to 0.309 by excluding additional 18 patients with grade-2 baseline lymphopenia, and further improved to 0.318 by excluding 84 grade-1 baseline lymphopenia patients. The percentage outliers were 2/3 (66.7%), 3/18 (11.1%), 2/84 (2.4%) and 1/640 (0.1%) for patients with baseline (Pre-RT) grade-3, grade-2, grade-1 and grade-0 lymphopenia, respectively. In patients with normal preRT PLC, the postRT PLC (or potential RIL) might be estimated by the linear regression of the post/preRT PLC ratio with EDIC, and specifically be computed as *PLC_postRT_* = *PLC_preRT_* × (0.89 – 0.16 × *EDIC*) (**Figure 1D**). Meanwhile, these data also indicated that patients with baseline lymphopenia before RT tended to have a different behavior.

**Figure 2:**
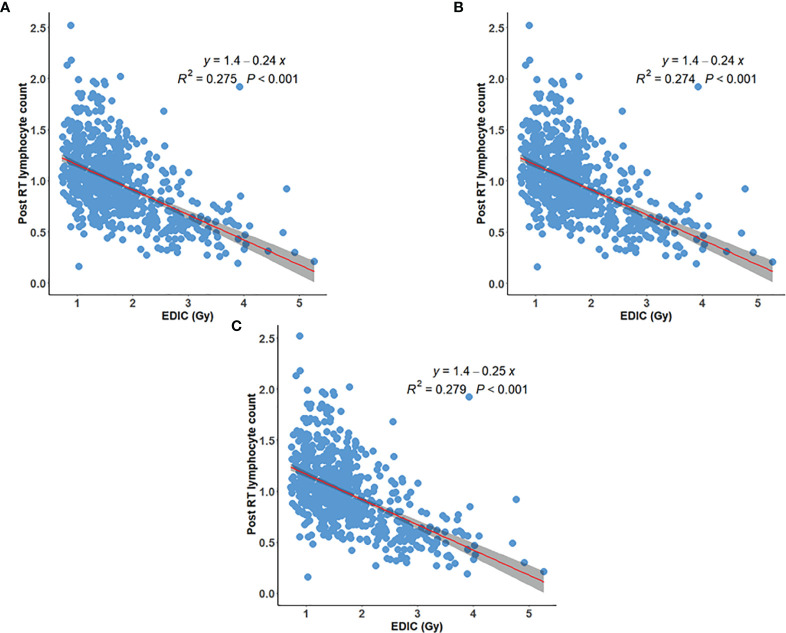
Inverse linear relationship between EDIC and postRT peripheral lymphocyte counts (PLC) for **(A)** all 735 patients; **(B)** 732 patients excluding 3 patients with grade 3 preRT lymphopenia; **(C)** 714 patients excluding additional 18 patients with grade-2 preRT lymphopenia.

The postRT PLC also decreased with increasing EDIC (R^2^ = 0.275, p<0.001) (**Figure 2A****)**, and its correlation was better than that of post/preRT PLC ratio to EDIC. However, the correlation did not improve when patients with preRT lymphopenia were excluded (**Figures 2B, C**). The correlation was better than that of the post/preRT PLC ratio to EDIC if all patients were considered, but it was not as good as that of the post/preRT PLC ratio to EDIC if patients with preRT lymphopenia were excluded.

**Figure 3:**
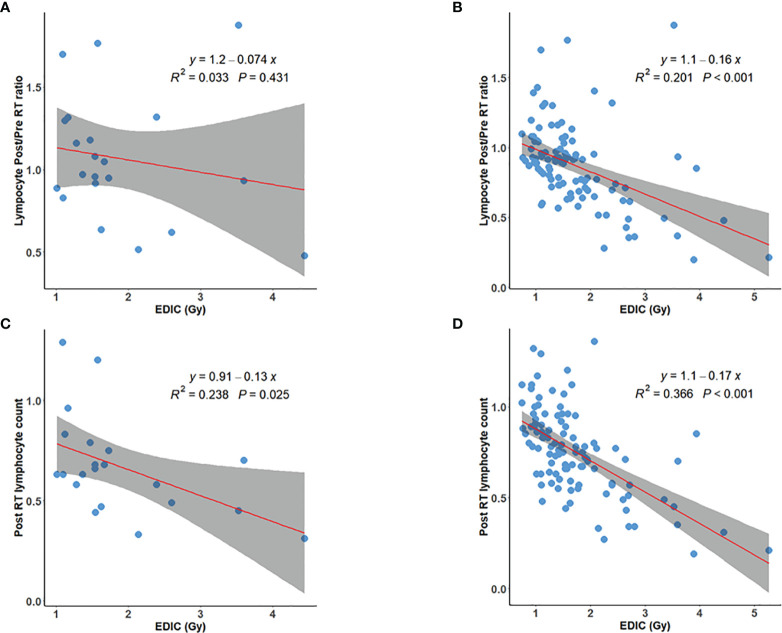
Post/preRT PLC ratio versus EDIC for **(A)** 21 patients with grade-2+ preRT lymphopenia; **(B)** 105 patients with grade-1+ preRT lymphopenia; PostRT PLC versus EDIC for **(C)** 21 patients with grade-2+ preRT lymphopenia; and **(D)** 105 patients with grade-1+ preRT lymphopenia.

We also compared the correlation of post/preRT PLC ratio to EDIC and postRT PLC to EDIC for patients with grade 1+ and grade 2+ pre-RT lymphopenia. As shown in **Figures 3A–D**, the correlation of postRT PLC to EDIC for patients with grade 1+ preRT lymphopenia was the best (R^2^ = 0.366, p<0.001). Therefore, for these patients with baseline lymphopenia, the postRT PLC (or potential RIL) might be approximated by linear regression of absolute postRT PLC with EDIC, and specifically be computed as *PLC_postRT_* = 1.1 – 0.17 × *EDIC* (**Figure 3D**). Again, all these data (**Figures 1**–**3**) indicate that overall, the post-RT lymphocytes decreased with increasing EDIC. However, the decreasing models may be different for patients with different pre-RT PLC status.

**Figure 4:**
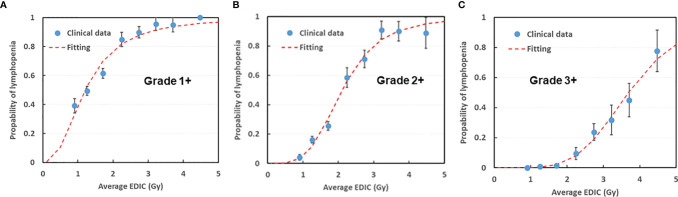
EDIC NTCP model for **(A)** Grade 1+, **(B)** Grade 2+ and **(C)** Grade 3+ postRT lymphopenia. Patients were divided into 8 groups with a 0.5 Gy increment in each group (<1, 1-1.5, 1.5-2, 2-2.5, 2.5-3, 3-3.5, 3.5-4, >4.5). NTCP, Normal Tissue Complication Probability. EDIC, Effective Dose to the circulating Immune Cells.

### RIL Normal Tissue Complication Probability (NTCP) Modelling

The NTCP versus mean EDIC based on the binned data was plotted in **Figures 4A–C** for Grade-1 and above, Grade-2 and above, and Grade-3 and above RIL respectively. The data were fitted with the NTCP model in Eq.1, with R^2^ = 0.96, 0.98 and 0.98, for Grade-1, Grade-2 and Grade-3 RIL, respectively. The D50 (dose of EDIC at 50% probability of having RIL) for EDIC was 1.2 (95%CI: 1.0-1.4), 2.1 (95%CI: 2.0-2.3) and 3.7 Gy (95%CI: 3.5-3.9), and the k value was 2.4 (95%CI: 1.8-3.2), 4.0 (95%CI: 3.1-5.3), and 4.9 (95%CI: 3.8-6.5), correspondingly. **Table 2** showed the results of model fitting of using binary data of each patient without binning, and Bootstrap validation for both binned data and binary data without binning. The binary data appeared to have quite consistent mean D50 and k values as the binned data, while its 95% CIs were narrower than that of the binned data. On the other hand, the binned data showed much better fittings (R^2^ Values) than that of the binary data of individual patient. The Bootstrap validation appeared to show very consistent data as the original data for both the binned data and binary data without binning.

**Table 2:** Parameters in NTCP modeling.

			Grade 1+	Grade 2+	Grade 3+
Binned data	Crude	D50 (Gy)	1.2 (95%CI: 1.0-1.4)	2.1 (95%CI: 2.0-2.3)	3.7 (95%CI: 3.5-3.9)
		k	2.4 (95%CI: 1.8-3.2)	4.0 (95%CI: 3.1-5.3)	4.9 (95%CI: 3.8-6.5)
		R^2^	0.96	0.98	0.98
	Bootstrap validation	D50 (Gy)	1.2 (95%CI: 1.1-1.3)	2.1 (95%CI: 2.0-2.2)	3.7 (95%CI: 3.3-4.4)
		k	2.4 (95%CI: 2.0-2.9)	4.0 (95%CI: 2.6-4.9)	5.5 (95%CI: 2.6-9.0)
		R^2^	0.93	0.96	0.94
Binary data without binning	Crude	D50 (Gy)	1.2 (95%CI: 1.1-1.3)	2.2 (95%CI: 2.1-2.3)	3.7 (95%CI: 3.6-4.0)
		k	2.1 (95%CI: 1.6-2.6)	3.9 (95%CI: 3.2-4.7)	4.4 (95%CI: 3.7-5.3)
		R^2^	0.13	0.3	0.27
	Bootstrap validation	D50 (Gy)	1.2 (95%CI: 1.1-1.4)	2.2 (95%CI: 2.0-2.3)	3.7 (95%CI: 3.4-4.4)
		k	2.1 (95%CI: 1.7-2.5)	3.9 (95%CI: 3.2-4.8)	4.4 (95%CI: 3.4-5.9)
		R^2^	0.12	0.3	0.27

The NTCP models for different grades of RIL could thus be expressed as:


for Grade-1 and above RIL (4a) $NTCP=1/\left[1+{\left(\frac{1.2}{EDIC}\right)}^{2.4}\right]$

for Grade-2 and above RIL (4b) $NTCP=1/\left[1+{\left(\frac{2.1}{EDIC}\right)}^{4.0}\right]$

for Grade-3 and above RIL (4c) $NTCP=1/\left[1+{\left(\frac{3.7}{EDIC}\right)}^{4.9}\right]$

## Discussion

This study of 735 patients at the first time in breast cancer demonstrated a significant correlation between EDIC and RIL, and that both post/preRT PLC ratio and postRT PLC decreased linearly (R^2^<0.4) with increasing EDIC. The post/preRT PLC ratio had a better correlation with EDIC in patients with normal preRT PLC, and the postRT PLC appeared to have a better correlation with EDIC in patients with preRT lymphopenia. Comparing to the numerical correlation by the linear regression, EDIC fitted better into the sigmoid-shaped NTCP model for RIL (R^2^>0.96). The EDIC value of 50% incidence of grade-1 RIL was 1.2 Gy, the corresponding EDIC to reach 50% of grade-2 and grade-3 RIL was 2.1 and 3.7 Gy, respectively.

The significance of EDIC for RIL in breast radiation makes biological sense and is supported by radiation physics rationales. Lymphocytes are produced in bone marrow and thymus, and then travel through blood circulation into various functional sites. Radiation can directly kill circulating immune cells as they pass through the irradiated field. EDIC is the estimation of equivalent dose to the circulating immune cells in blood contributed by the irradiation of the blood-containing organs in thoracic radiation according to the mean dose to these organs (such as MLD, MHD and ITDV). In addition, the radiation dose fractionation effect is also considered in the EDIC model (25). EDIC has been reported to be associated with RIL in other thoracic malignancies such as lung and esophageal cancers (15, 25–27). As a type of thoracic cancers, breast cancer is similar to the NSCLC and esophageal cancer in terms of that its main irradiated blood-containing organs are lung and heart. Therefore, the EDIC model may be also applicable in breast cancer. However, the irradiation pattern or dose distribution in these organs may be quite different for breast cancer in comparison to that of the NSCLC and esophageal cancer. The irradiated lung and heart volumes in breast cancer are usually much smaller. According to the EDIC model derivation (25), the contribution of a blood-containing organ (such as lung) to EDIC is approximately expressed as B%*Mean Organ Dose (MOD) when A% is larger than 20% and fraction number is larger than 20, where MOD is the mean organ dose, A% is the percentage blood-current that flow into the organ, B% is the percentage blood-volume that present within the organ. This expression was derived based on the assumption that the blood flow through the organs in a serial pattern, and should be applicable for any blood-containing organs with any non-uniform dose distributions. The lung is composed of 5 lobes and each lobe may be approximately considered as having a serial blood-flow pattern with A%=20%. From the physics model perspective, the EDIC model can be applied in breast cancer radiation. Indeed, significance of EDIC on both postRT PLC and RIL of this study support the validity of this model in breast cancer.

It is interesting to note that when a patient had a normal preRT PLC, the post/preRT PLC ratio had better correlation with EDIC. This is consistent with the theory that when the preRT PLC is normal, the killing of circulation lymphocytes in blood by radiation plays the most important role in the reduction of PLC. However, 14.3% patients had preRT lymphopenia in this study, which was reported to be related with prior chemotherapy in our another study of 1012 patients with early or locally advanced breast cancer (28). Therefore, we also explored the relationship of EDIC and lymphopenia in this population. When a patient had preRT lymphopenia, the absolute postRT PLC was better associated with EDIC. This is consistent with previous report that when the preRT PLC is low, the regeneration of new lymphocytes from other parts of lymphatic system begins to play a role (12). This regeneration of new lymphocytes weakens the association between EDIC and post/preRT PLC ratio. The lower the preRT PLC, the larger the deviation from the correlation of EDIC and post/preRT PLC ratio. For 3 patients with grade-3 preRT lymphopenia, 2 of them were outliers, while 3 over 18 patients were outliers for grade-2 preRT lymphopenia, and 2 over 84 patients were outliers for grade-1 preRT lymphopenia in this study.

It is important to note that this study also provided a NTCP model of RIL based solely on EDIC. The NTCP model of each grade of lymphopenia fits well with the sigmoid-curved dose toxicity relationship, using EDIC for both binned data or binary data for each patient without binning. These two approaches achieved quite consistent results, and bootstrap validation also confirmed the similar results. These data suggest that EDIC is likely a true key dosimetric parameter, or at least a good surrogate of the true dosimetric parameter that directly impacts the RIL. EDIC might be used as a reference for clinician in dose prescription and treatment planning. With the guide of this EDIC model in lymphopenia, we may improve our treatment to limit RIL. For example, adjustment of beam energies, directions and number of beams may be able to reduce EDIC and hence limit RIL. In addition, according to the EDIC derivation (25), reducing the radiation fraction number *n* may also reduce EDIC through the term of (*n*/45)^1/2^, which is contributed by blood-containing organs with a relatively small A% (such as A% <25%). Thus, hypofractionated treatments such as stereotactic body radiation therapy (SBRT) may reduce EDIC. Indeed, SBRT was reported to be associated with significantly less RIL than conventionally fractionated RT in locally advanced pancreatic cancer and breast cancer (18, 29, 30). Hypofractionated radiation in this study was also found to have less risk of RIL (**Table 1**).

This is the first study to validate the association of EDIC and RIL in patients with breast cancer. However, it should be noted that several other dosimetric factors were also reported to be significantly associated with RIL. These factors include lung V5 and heart V50 in lung cancer, brain V25 in glioma, and mean whole body dose in esophageal cancer (9, 12, 13, 31–33). In our prior study, lung V5 and volume modulated arc therapy rather than three-dimensional conformal technique were also risk factors for severe RIL (16). Although EDIC is a combination of several dosimetric parameters, it may not be the best model. A more comprehensive model with an optimal combination of other dosimetric parameters (such as lung V5) and likely including immune organs like bone marrow and thymus may turn out to be a better predictor than the EDIC. Adding other clinical factors may further improve the prediction of RIL in breast cancer. Further study is needed to determine these parameters and the best combination of them for better prediction. From a theoretic view point, EDIC is just an approximation of the dose to the immune cells in the circulating blood. The dose to the lymphatic stations, bone marrow and other lymphatic structures may also affect RIL. The regeneration of new lymphocytes may complicate the prediction. It should also be noted that the survival of this study is not mature yet. Future studies will be performed to explore the association of EDIC and other dosimetric parameters with the long-term treatment outcomes of patients.

## Conclusions

This study demonstrated that EDIC was significantly associated with radiation induced lymphopenia (RIL) in breast cancer. Using EDIC as the dose variable, the risk of RIL can be predicted nicely by a conventional NTCP model. The corresponding EDIC to induce 50% of grade-1, grade-2 and grade-3 RIL was 1.2, 2.1 and 3.7 Gy, respectively. Should it be validated by external datasets, these number may be used as reference to guide radiation plan optimization and improve survivals for patients.

## Data Availability Statement

The raw data supporting the conclusions of this article will be made available by the authors, without undue reservation.

## Ethics Statement

The studies involving human participants were reviewed and approved by the University of Hong Kong-Shenzhen Hospital (# 2019 098). Written informed consent for participation was not required for this study in accordance with the national legislation and the institutional requirements.

## Author Contributions

FC: primary investigator: overall study hypothesis, study design, data collection, data analysis, result interpretation, manuscript writing and final manuscript approval; J-YJ and TH: study design, physics modeling, data analysis, manuscript writing and final manuscript approval; HZ, LM, and PF: various parts of statistical analysis, results interpretation and final manuscript approval; YH, FY, QC, YZ: clinic data collection and final manuscript approval; YN: radiation dosimetric factors collection and final manuscript approval; HJ, WW, LY, and ZX: results interpretation and final manuscript approval; F-MK: study idea, study hypothesis/design, data quality control, data analysis, result interpretation, detailed manuscript preparation and final manuscript approval. All authors contributed to the article and approved the submitted version.

## Funding

This project was partially supported by Shenzhen Key Medical Discipline Construction Fund (No. SZXK014), Shenzhen Science and Technology program (Grant No: KQTD20180411185028798), Health Commission of Guangdong Province, China (A2021114) and a Varian Medical System research grant.

## Conflict of Interest

The authors declare that the research was conducted in the absence of any commercial or financial relationships that could be construed as a potential conflict of interest.

## Publisher’s Note

All claims expressed in this article are solely those of the authors and do not necessarily represent those of their affiliated organizations, or those of the publisher, the editors and the reviewers. Any product that may be evaluated in this article, or claim that may be made by its manufacturer, is not guaranteed or endorsed by the publisher.

## Supplementary Material

Supplementary Figure 1 Study population profile. As shown, a total of 735 patients with breast cancer were enrolled in this study.

## Abbreviations

EDIC, effective dose to the circulating immune cells; RIL, radiation induced lymphopenia; RT, radiotherapy; PLC, peripheral lymphocyte counts; NSCLC, non-small cell lung cancer; LYM, lymphocytes; Nadir-PLC, the lowest peripheral lymphocyte counts during radiation; PreRT PLC, peripheral lymphocyte counts before radiation; OAR, organ at risk; CTCAE, Common Terminology Criteria for Adverse Events; EUD, equivalent uniform dose; MLD, mean lung doses; MHD, mean heart dose; ITDV, integral dose or integral total dose volume; NTCP, Normal Tissue Complication Probability; OS, overall survival; LPFS, local progression-free survival; CRT, chemoradiotherapy; PFS, progression free survival; DMFS, distant metastasis free survival; ESCC, esophageal squamous cell cancer; SBRT, stereotactic body radiation therapy; MOD, Mean Organ Dose; V5 (20), relative volume receiving more than 5Gy (20Gy).
