# Supplementary figures and images for: Radiation Induced Lymphopenia Is Associated With the Effective Dose to the Circulating Immune Cells in Breast Cancer
Source: Front Oncol. 2022 Apr 28;12:768956. doi: 10.3389/fonc.2022.768956 (PMC9118537; doi:10.3389/fonc.2022.768956)

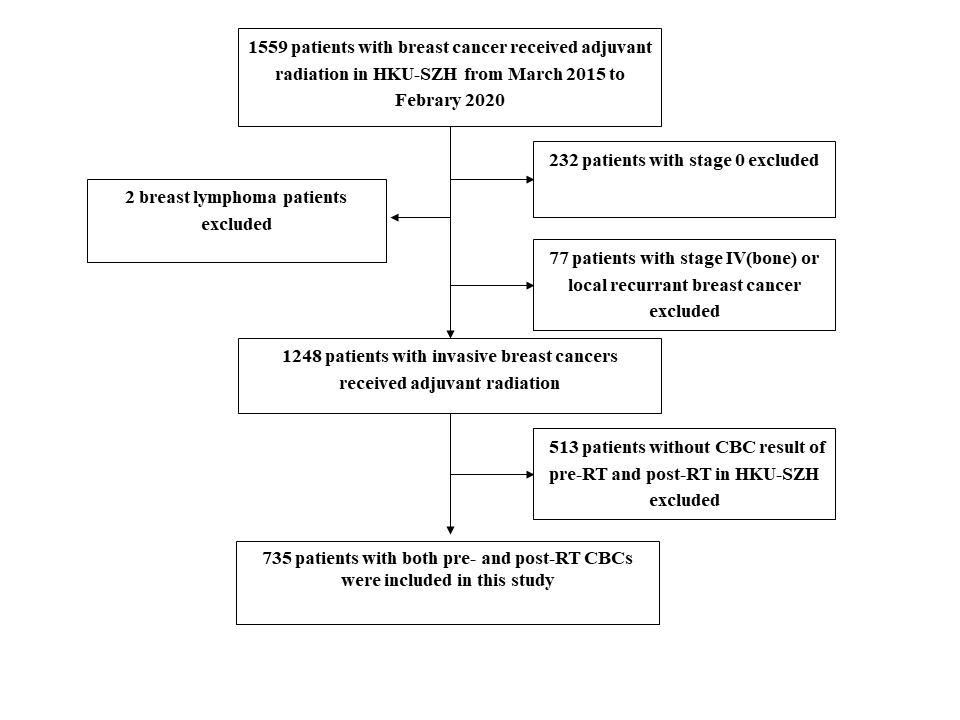

Supplement: Supplementary Figure 1 — Study population profile. As shown, a total of 735 patients with breast cancer were enrolled in this study. [file Image_1.tif]
